# Risk assessment of obstructive sleep apnea syndrome in pediatric patients with vitamin D deficiency: A questionnaire-based study

**DOI:** 10.1097/MD.0000000000004632

**Published:** 2016-09-30

**Authors:** Gamze Ozgurhan, Aysel Vehapoglu, Oznur Vermezoglu, Rabia Nur Temiz, Asuman Guney, Bulent Hacihamdioglu

**Affiliations:** a Department of Pediatrics, Suleymaniye Maternity and Children's Training and Research Hospital; b Department of Pediatrics, Bezmialem Vakıf University, Faculty of Medicine, İstanbul, Turkey.

**Keywords:** 25-hydroxyvitamin D, body mass index, sleep apnea, vitamin D deficiency, z-score

## Abstract

The aim of the following study is to evaluate the risk of obstructive sleep apnea syndrome (OSAS) in subjects with vitamin D deficiency.

Prospective and comparative study.

We enrolled 240 subjects into the study. The participants were divided into 2 groups based on 25-hydroxyvitamin D (25[OH]D) levels: low level of 25(OH)D (<20 ng/mL) group (n = 120) and control (>20 ng/mL) group (n = 120). Subjects were classified as being at a high or low risk of developing OSAS by using the Berlin Questionnaire. Risk of developing OSAS, gender, age, and body mass index (BMI) z-score were assessed by comparing the low level of 25(OH)D group and control group.

No statistically significant difference was observed between the low level of 25(OH)D group and control group in terms of gender, age, and BMI z-score distributions; *P* = 0.323, *P* = 0.387, and *P* = 0.093, respectively. There were 24 subjects with high risk of developing OSAS in 2 groups (17 subjects in the low level of 25[OH]D group and 7 subjects in the control group). In the low level of 25(OH)D group, the risk of developing OSAS was found to be significantly higher than the control group (*P* = 0.030). BMI z-score was found significantly higher in high-risk groups than low-risk groups (*P* = 0.034 for low-level 25[OH]D group and *P* = 0.023 for control group).

The findings revealed that low level of 25(OH)D increases the risk of developing OSAS.

## 1 Introduction

Obstructive sleep apnea syndrome (OSAS) is a common disorder in childhood affecting up to 3% to 4% of all children.^[1]^ The most important demographic risk factors for this disease are obesity, age, and male gender.^[2,3]^ It has a wide spectrum of important clinical symptoms, including excessive daytime sleepiness, cognitive dysfunction, cardiovascular diseases, respiratory system infections, and metabolic dysfunction.^[2–9]^

Recently, it has become clear that vitamin D has many effects and plays an important role in a broad range of organ functions. It is also known that vitamin D deficiency (VDD) is a common worldwide condition. Therefore, it is essential to gain a deep understanding of the potential effects of VDD. VDD has been associated with similar metabolic disturbances as OSAS.^[10–11]^ Although the nonskeletal consequences of VDD are not well understood, many evidence suggest that low levels of 25-hydroxyvitamin D (25[OH]D) have been associated with increases in the frequency and severity of metabolic dysfunction, obesity, and cardiovascular diseases, as well as increased incidence of respiratory tract infections.^[12–18]^

Nocturnal polysomnography (PSG) is required to diagnose, exclude OSAS, or assess its severity. PSG is the gold standard method for the diagnosis of OSAS but is not suitable for screening. The sleep questionnaire is easily applicable and predicts polysomnographic results to an extent that is useful for clinical research.

In this study, we investigate the risk of OSAS by using a sleep questionnaire in children with VDD. We hypothesize that risk of OSAS is higher in children with reduced vitamin D.

## 2 Materials and methods

This prospective and comparative study was performed at the Suleymaniye Maternity and Children's Training and Research Hospital. The study was conducted according to the principles of the Declaration of Helsinki and was approved by the local ethics committee. The subjects and their parents were informed, and they provided written informed consent.

### 2.1 Study groups

Subjects were divided into 2 groups according to levels of 25(OH)D, a low-level 25(OH)D group (<20 ng/mL) and a control group (>20 ng/mL). The low-level 25(OH)D group was matched for age, sex, and body mass index (BMI) z-score with the control group.

Each participant underwent a comprehensive assessment including a review of medical history, physical examination, and height and weight measurements. The study included the subjects who were healthy individuals without any chronic disease such as chronic renal failure, malabsorption, nutritional rachitis, and adenotonsillar disease. There was no subject with a history of OSAS in both groups. No participant reported taking calcium (Ca) or multivitamin supplements. Furthermore, all participants needed to be 7 to 14 years old.

### 2.2 Laboratory

25(OH)D plasma level was assessed at UniCel DxI 800 autoanalyzer using access immunoassay system 25(OH)D (Beckman Coulter, O’Callaghans Mills, Ireland) kit with chemilluminescence method. Ca and phosphor levels were assessed at Cobas c 501 autoanalyzer using Ca2 and Phos2 (Roche/Hitachi, Berlin, Germany) kits with photometric method. Alkaline phosphatase (Alk Phos) plasma level was assessed at Cobas c 501 autoanalyzer using ALP2 (Roche/Hitachi) kit with calorimetric method. Normal values were 8.4 to 10.2 mg/dL for Ca, 3.1 to 5.3 mg/dL for phosphor and <300 U/L for Alk Phos.

### 2.3 Berlin Questionnaire

The Berlin Questionnaire (BQ) was completed during face-to-face interviews with subjects and their parents in this study. The participants were questioned primarily about their OSAS history, and the BQ was used to evaluate the risk of developing OSAS. Using the BQ, which has 3 parts, subjects were reliably classified as being at a high or low risk of developing OSAS. The criteria were snoring behavior, daytime sleepiness, and high blood pressure or obesity. Obesity was defined with BMI z-score >2. It is calculated by dividing weight (kg) by square of height (m) and then transformed to an age- and sex-specific z-score. If 2 of these 3 conditions were present, subjects were classified as being at a high risk of developing OSAS. The questionnaires were analyzed by the same specialist (GO), and the observer was masked to control group.

### 2.4 Data analysis

All statistical analyses were performed using the Statistical Package for Social Sciences (SPSS) version 20.0 for Windows (SPSS, Chicago, IL). The 1-sample Kolmogorov–Smirnov test was used to evaluate the distribution characteristics of variables. Demographic and clinical characteristics of low 25(OH)D and control groups were compared using the Mann–Whitney *U* test for continuous variables and the χ^2^ test for categorical variables. Differences were considered statistically significant for *P* values less than 0.05.

## 3 Results

The study included 240 subjects: 120 subjects in the low-level 25(OH)D group and 120 subjects in the control group. No statistically significant difference was observed between the 2 groups in terms of gender, age, and BMI z-score distributions; *P* = 0.323, *P* = 0.387, and *P* = 0.093, respectively. High risk of developing OSAS was significantly higher in the low-level 25(OH)D group than in the control group (*P* = 0.030). The demographic and clinical characteristics of both groups are shown in Table 1.

**Table 1:**
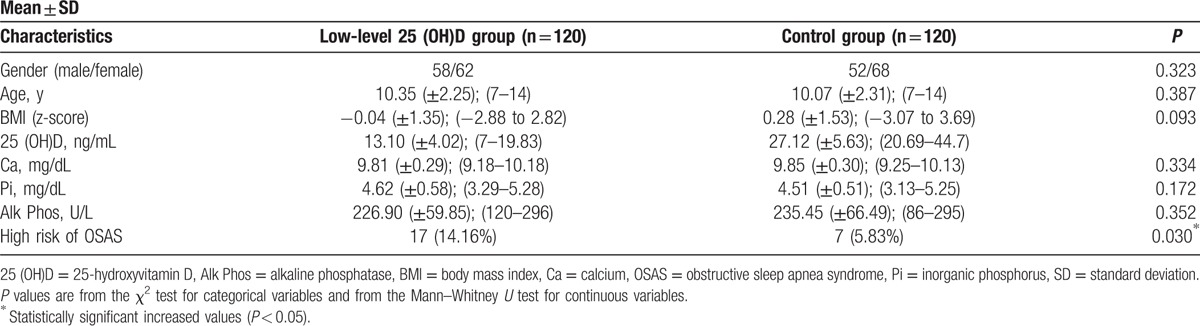
The demographic and clinical characteristics of low-level 25(OH)D and control groups.

When 2 groups were classified as being at a high or low risk of developing OSAS, there were no significant difference for gender and age between high- and low-risk groups. In high-risk groups, BMI z-score was found to be significantly higher than low-risk groups (Table 2).

**Table 2:**
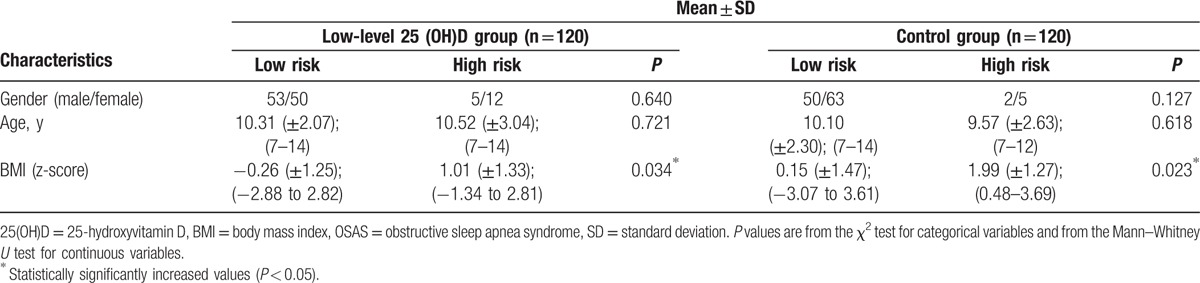
Characteristics of risk of developing OSAS in low-level 25(OH)D and control groups.

## 4 Discussion

In this study, we assessed the risk of developing OSAS in children with VDD. Although there is no study in the literature similar to ours, there are many studies that report VDD in adult or children patients with OSAS,^[19–22]^ and results of these studies are correlated with those of ours. This study has shown that the risk of developing OSAS as determined by the BQ was found to be statistically significantly higher in low-level 25(OH)D group when compared with the control group (*P* = 0.030). The percentage of patients at a high risk of developing OSAS was 14.16% for the low-level 25(OH)D group and 5.83% for the control group.

Obesity is one of the best defined risk factors for OSAS. BMI is strongly associated with OSAS prevalence.^[23]^ On the other hand, there is an inverse association between BMI and 25(OH)D. In many studies, obesity has been found to be common in patients with OSAS.^[22–25]^ In our study, median BMI z-score values were within normal range in low-level 25(OH)D and control groups (*P* = 0.093). But in the patients with high risk of developing OSAS, BMI z-score was found to be significantly higher than the patients with low risk of developing OSAS in both the low-level 25(OH)D and control groups.

Many studies show that the decline in 25(OH)D level is associated with risk of OSAS in children. Recently, functional roles of 25(OH)D have been unveiled. Vitamin D insufficiency is widely prevalent and leads to many health problems beyond bone disease, such as nonspecific pain and noninflammatory skeletal myopathy, which may disrupt sleep and directly cause daytime sleep impairment. Emerging lines of evidence suggest that low vitamin D levels increase the risk for autoimmune disease, chronic rhinitis, tonsillar hypertrophy, cardiovascular disease, metabolic syndrome, and diabetes. It was claimed that persistent inadequacy of vitamin D may also increase the risk for obstructive sleep apnea via promotion of adenotonsillar hypertrophy, airway muscle myopathy, and/or chronic rhinitis.^[12,18,20,26–30]^ In this study, we only show that OSAS risk may be high in children with VDD but we could not evaluate pathogenic link between the two. Also in this study, we define the VDD according to the cutoff value of bone health, but optimal vitamin D level for nonskeletal organs and tissues is not known.

Vitamin D is essential for an appropriately responsive immune system. Inadequate vitamin D clinically results in increased risk of infections and inflammation involving the upper and lower airway. This may increase the risk for OSAS. Also vitamin D contributes to sleepiness through such central signaling, and inflammatory mediators are likely to be involved. The sleep-regulating substances, tumor necrosis factor-alpha and IL-1, both exhibited inverse relationships with 25(OH)D. More information is needed regarding the link between circulating 25(OH)D and OSAS.^[31–34]^ However, our study is not focused on the causal link between these conditions.

25(OH)D was inversely associated with numerous OSAS parameters as documented with PSG. This inverse correlation has been reported by Bozkurt et al.^[21]^ They pointed out that those patients with more severe OSAS polysomnographic indices tended to present lower vitamin D levels. In another study, 25(OH)D was associated with OSAS according to multivariate logistic regression analysis.^[25]^

Although the BQ for OSAS risk evaluation is a widely used and reliable test, this research was restricted by some limitations. First, the evaluation of OSAS risk was done only with BQ rather than an additional confirmation with PSG. Second, this study has a relatively small sample size, and a larger study population is needed to validate these findings. Even with these limitations, this is the first study evaluating risk of OSAS in VDD cases.

In summary, low level of vitamin D is common in childhood and may be the cause of or a contributor to some disorders such as OSAS. Obstructive sleep apnea syndrome reduces quality of life by affecting central nervous system, cardiovascular system, metabolic system, and somatic growth. In this study, we have concluded that there is a correlation between low vitamin D levels and risk of OSAS. To prevent potential negative impacts of OSAS and decrease its morbidity, there is need for broader and deeper follow-up studies.
